# Interments in the City and Liberties of Philadelphia, from the 29th of May, to the 5th of June, 1841

**Published:** 1841-06-12

**Authors:** 


### HEALTH OF THE CITY.

INTERMENTS *in the City and Liberties of Philadelphia, from the 29th of May, to the 5th of June,* 1841.

| Diseases. | Adults. | Children. | Diseases. | Adults. | Children. |
|---|---|---|---|---|---|
| Abscess of Lungs, | 1 | 0 | *Brought forward,* | 40 | 33 |
| Angina Pectoris, | 0 | 1 | Jaundice, | 0 | 1 |
| Asphyxia, | 0 | 1 | Marasmus, | 1 | 3 |
| Croup, | 0 | 3 | Malformation, | 0 | 1 |
| Congestion of brain, | 0 | 2 | Measles, | 0 | 6 |
| Consumption of the lungs, | 16 | 4 | Mania a potu, | 1 | 0 |
| Convulsions, | 0 | 1 | Old age, | 1 | 0 |
| Diarrhœa, | 1 | 0 | Ossification of Arteries, | 1 | 0 |
| Dropsy, | 1 | 0 | Scirrhus, | 1 | 0 |
| —— Abdominal, | 1 | 0 | Scrofula, | 1 | 1 |
| —— head, | 0 | 2 | Small pox, | 0 | 1 |
| —— breast, | 1 | 1 | Still-born, | 0 | 6 |
| Disease of Kidneys, | 1 | 0 | Summer Complaint, | 0 | 2 |
| Drowned, | 1 | 0 | Syphylis, | 1 | 0 |
| Debility, | 0 | 4 | Unknown, | 3 | 1 |
| Effects of Laudanum, | 1 | 0 | | — | — |
| Erysipelas, | 2 | 0 | Total, 105— | 50 | 55 |
| Enlargement of Heart, | 1 | 1 | | | |
| Effusion of Lungs, | 1 | 0 | | | |
| Fever, | 0 | 1 | | | |
| —— remittent, | 0 | 1 | | | |
| —— Typhus, | 2 | 0 | | | |
| —— Typhoid, | 1 | 1 | | | |
| Hæmorrhage, | 1 | 0 | | | |
| Inflammation of the Brain, | 2 | 4 | | | |
| —— Bronchi, | 0 | 2 | | | |
| —— Lungs, | 3 | 3 | | | |
| —— Stomach, and Bowels, | 1 | 0 | | | |
| —— Bowels, | 0 | 1 | | | |
| —— Liver, | 1 | 0 | | | |
| | — | — | | | |
| *Carried forward,* | 40 | 33 | | | |

Of the above, there were under 1 year, 24

| | |
|---|---|
| From 1 to 2 | 9 |
| 2 to 5 | 13 |
| 5 to 10 | 4 |
| 10 to 15 | 2 |
| 15 to 20 | 3 |
| 20 to 30 | 17 |
| 30 to 40 | 11 |
| 40 to 50 | 6 |
| 50 to 60 | 3 |
| 60 to 70 | 4 |
| 70 to 80 | 7 |
| 80 to 90 | 2 |
| 90 to 100 | 0 |
| Total, | 105 |

Of the above there were 8 from the almshouse, 20 people of colour, and one from the country, which are included in the total amount.

